# Evolving perceptual biases for antisynchrony: a form of temporal coordination beyond synchrony

**DOI:** 10.3389/fnins.2015.00339

**Published:** 2015-09-30

**Authors:** Andrea Ravignani

**Affiliations:** ^1^Artificial Intelligence Lab, Vrije Universiteit BrusselBrussels, Belgium; ^2^Sensory and Cognitive Ecology Group, Universität RostockRostock, Germany

**Keywords:** evolution of communication, group, animal behavior, time perception, social behavior, sexual selection, rhythm, evolution of music

## Overview

Many organisms coordinate their group behavior in time. On a short timescale, group vocalizations, movements or visual displays can exhibit temporal interdependence. Synchronous behavior has received significantly more attention than all other forms of animal coordination. Antisynchrony (i.e., perfect alternation) is produced in nature, but only recently perceptual biases toward antisynchrony were independently found in human infants and fiddler crabs. Here, these unrelated experiments are linked and inserted into a broader quantitative framework. Future comparative research should encompass perception of other forms of coordination across species and explanatory levels, toward an integrative neuro-evolutionary framework of temporal coordination.

## Synchrony: one among many forms of temporal interaction

**Figure 1:**
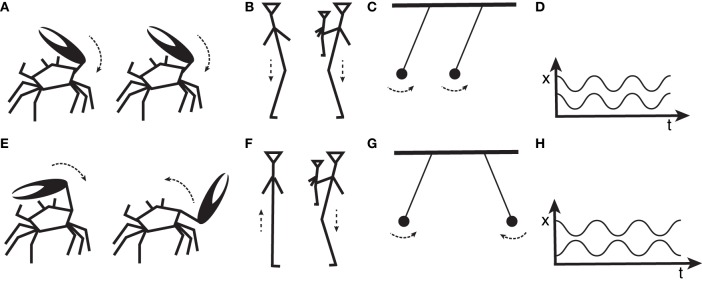
**Synthetic representation of synchronous (top row) and antisynchronous (bottom row) coordinated behaviors**. Male robotic fiddler crabs wave their larger claw in **(A)** synchrony or **(E)** antisynchrony (Kahn et al., 2014). Similarly, two human adults, one holding an infant, move up, and down to music in **(B)** synchrony, as if each was dancing with her own mirror image or **(F)** antisynchrony, so that one bends her knees while the other stands straight, and vice-versa (Cirelli et al., 2014). Physical oscillators, like pendulums, can resonate at the same frequency; in addition, **(C)** their phase delay can be 0, making them synchronous, or **(G)** half of the oscillatory period, namely π, corresponding to antisynchrony (Strogatz and Stewart, 1993). Events happening in time can be represented graphically by plotting the displacement x–be it the movement of a human leg, a crab's claw or a pendulum–over time t. Plotting time series in this way makes periodic phenomena readily recognizable by their regularly repeating oscillations. In particular, **(D)** synchronous phenomena produce similar sinusoidal waves which can be graphically overlapped, while **(H)** antisynchronous phenomena also produce similar waves, which can however only be overlapped by (phase) shifting one of the sinusoids over time (leftwards or rightwards). Key findings and research efforts to date have been focusing on one particular coordination mode: synchrony (Buck and Buck, 1968; Tuttle and Ryan, 1982; Winfree, 1986; Ermentrout, 1991; Grafe, 1999; Patel et al., 2009; Hasegawa et al., 2011; Merchant et al., 2011; Hattori et al., 2013; Aihara et al., 2014; Fuhrmann et al., 2014; Gamba et al., 2014; Ravignani, 2014; Ravignani et al., 2014a,b; Large and Gray, 2015; Yu and Tomonaga, 2015). However, synchronous behavior is only one outcome of coordinated interactions (Morris et al., 1978; Haimoff, 1986; Grafe, 1999; Bermejo and Omedes, 2000; Yosida and Okanoya, 2005; Mann et al., 2006; Brumm and Slater, 2007; Yosida et al., 2007; Hall, 2009; Ravignani et al., 2013; Aihara et al., 2014; ten Cate, 2014; Hattori et al., 2015); for instance, several species show antiphonal (constant lag) coordination (Sismondo, 1990; Yosida and Okanoya, 2005; Mann et al., 2006; Yosida et al., 2007; Inoue et al., 2013).

Synchrony, when two or more events take place at exactly the same time, is the most ordered form of temporal coordination (Figures 1A–D, top row). Crickets chorus in synchrony, fireflies flash likewise, all with millisecond accuracy (Buck and Buck, 1968; Buck, 1988; Sismondo, 1990; Hartbauer and Römer, 2014). Synchrony does not entail individual intentions to coordinate but often arises as an epiphenomenal by-product of selfish behavior (Greenfield and Roizen, 1993): Individuals want to be noticed. The ecological, behavioral, and neural bases underpinning synchronous behavior have been intensively explored and are increasingly understood (Greenfield et al., 1997; Hartbauer et al., 2005; Fitch, 2015; Iversen et al., 2015).

Yet, synchronous behavior is only one solution to well-coordinated interactions. Many degrees of coordination separate synchrony, like an orchestra in unison, from independent behavior, like several musicians each rehearsing alone (Strogatz and Stewart, 1993; McNeill, 1997). However, perception of all forms of non-synchronous coordination remains mostly unexplored.

## Perceptual biases: what catches the eye?

In general, animals show perceptual biases toward particular physical patterns. Here, bias means a predilection of a species' sensory system for particular features, which are perceptually conspicuous to the species. Signallers draw receivers' attention by sending signals; often these signals simply exploit receivers' perceptual biases, rather than advertise good genes and fitness of the signallers (Ryan, 1998). For instance, several animals exhibit colorful fur or plumage, and simultaneously their visual perception is driven toward bright colors. In several animal species, a bias toward red/yellow colors was useful for e.g., finding ripe fruits and was likely also co-opted as a mate selection device.

A similar logic can be applied, possibly for the first time, to perception of group coordination in the temporal domain^1^. Which sensory biases drive animals toward rhythmic coordination beyond synchrony? Convergent results from child development, animal behavior, and dynamical systems suggest antisynchrony may provide a first answer (Figures 1E–H). *Antisynchrony* is the closest alternative to synchrony in physical terms (Figures 1C–G). Perceptually, antisynchrony consists in perfect alternation, as in a walking march. In other words, a constant time period separates pairs of antisynchronous movements. Two new experiments in unrelated disciplines simultaneously show that organisms are driven toward the same temporal coordination pattern. Both human infants and crabs exhibit, among others, a perceptual bias toward antisynchrony.

## Crabs are driven toward antisynchrony

Male fiddler crabs (*Uca mjoebergi*) have one claw larger than the other, which they wave to attract females (Backwell et al., 1998). Each crab finely times its movements depending on the female audience and male competitors. Male fiddler crabs often end up waving in synchrony (Figure 1A; Backwell et al., 1998).

Ingenious methodologies and carefully designed experiments have elucidated why temporal interdependence should arise when individual males compete to be noticed by females. Robotic replicas of male crabs were programmed to simulate a number of temporal coordination scenarios, waving in synchrony, antisynchrony, etc. Actual crab females were then tested on their willingness to approach individual robotic crabs, or group of crabs, in different coordination patterns (Reaney et al., 2008). Since individual timing influences perceived attractiveness, females' choices reveal female perceptual biases and preferences for particular temporal patterns. When presented with two groups of male crabs, one waving in synchrony, the other in antisynchrony, females were equally likely to choose between the two groups (Reaney et al., 2008). Female crabs were also tested on their willingness to approach individual robotic crabs *within* a male group. Crucially, the crab waving in antisynchrony with the rest of the group (Figure 1E) was one of the favorite among different timing coordination conditions (Kahn et al., 2014).

Movement alternation granted by antisynchrony might be particularly effective to obtain females' attention. Antisynchrony—a previously neglected mode of coordination—was finally shown to be as conspicuous as synchrony in a non-human animal.

## Antisynchrony triggers prosociality in human infants

Research on human evolution and behavior has profited in the last decades from integration of ethology and human developmental studies (Fitch, 2015; Trainor, 2015). Studying behavioral traits in culturally-naïve infants, and comparing them with similar behaviors in other species, niches and environments, can shed light on human evolution (Hagen and Hammerstein, 2009; Trainor, 2015). It is hence fortunate that cognitive neuroscientists, mutually unbeknown to animal behavior researchers, have also just found biases for antisynchrony in human infants. Temporal movement coordination in human adults has a well-known social role (Cirelli et al., 2014), and temporal coordination and sociality have been usually investigated during synchronous interactions. In particular, perceptual and attentional biases toward movement synchrony are present in humans, and synchronous interactions increase prosocial behaviors, such as cooperation, social cohesion, etc (Hove and Risen, 2009; Miles et al., 2009; Wiltermuth and Heath, 2009; Kirschner and Tomasello, 2010; Manson et al., 2013; Cirelli et al., 2014). When adults are asked to tap together, they soon fall into synchrony or antisynchrony (Knoblich et al., 2011).

Recent experiments in human infants started clarifying the developmental pathways of perceptual biases for coordination, adding antisynchrony to the repertoire. 14-month-old infants were held by an experimenter and exposed to different interpersonal coordination scenarios. In some of those, the experimenter would move the infant up and down in synchrony (Figure 1B), antisynchrony (Figure 1F) or asynchrony (i.e., random timing) with another adult moving to music. After being bobbed in synchrony and antisynchrony with an adult, infants were more prosocial than after asynchronous movements (Cirelli et al., 2014). In particular, infants exhibited more spontaneous, but not delayed, helping behavior: synchrony and antisynchrony affected early stages of infants' sensory perception, but ceased to influence social behavior as soon as infants exchanged gaze or vocalizations with an adult. This suggests that Cirelli et al.'s experimental setup (i) tapped into early, possibly evolutionary ancient neuroethological traits (Trainor, 2015) dating to our last common ancestor with great apes, or earlier (Fitch, 2009; Giacoma et al., 2010; Hagmann and Cook, 2010; Dunbar, 2012; Gamba et al., 2014; Dufour et al., 2015; Large and Gray, 2015; Yu and Tomonaga, 2015), hence their results could help uncover the phylogenetic bases of rhythm; (ii) engaged human participants' subcortical brain structures [such as basal ganglia, usually involved in perception of rhythmic patterns (Grahn and Brett, 2007; Kotz and Schmidt-Kassow, 2015)], again suggesting that preferences for (anti)synchrony are likely to be found in other animals due to common ancestry.

## Human temporal coordination: evolution and functions

In human evolutionary history, refined temporal coordination and perception abilities might predate the origins of music and speech (Bryant, 2014; Ravignani et al., 2014c). Finely coordinated dance and music might have initially arisen as a social device, possibly as a signal of group cohesion (Merker, 2000; Hagen and Bryant, 2003; Merker et al., 2009; Dunbar, 2012). Now, every signaling system relies on a perceptual repertoire, which can be exploited for communication: biases toward particular temporal coordination patterns, like synchrony and antisynchrony, could have offered such fertile perceptual substrate for a joint group signaling system. The hypothesis that (anti)synchrony mediated group coordination and music origins is supported by another “evolutionary leftover” found in the auditory domain. Modern humans prefer syncopated music (Fitch and Rosenfeld, 2007; Keller and Schubert, 2011), which also provides a sense of groove (urge to move rhythmically, Janata et al., 2012). Crucially, syncopated rhythms in music often correspond to musical notes in antisynchrony with the underlying beat.

## A common perceptual bias for antisynchrony?

Infants and fiddler crabs are driven toward the same form of mild asynchrony. A basic perceptual bias for a simple coordination mode—antisynchrony—might have been a precursor for behaviors as different as prosociality and mate selection. (This would be analogous to a single physical trait exapted by two species for different usages, e.g., humans walk on legs, harbor seals swim with hind-flippers, and both limb types evolved from the back legs of our quadruped ancestor). Why would specifically antisynchrony be exapted, and not other coordination modes? Antisynchronous movements reunite two conditions: they follow periods of no waving and, by definition, are uncluttered by other synchronous movements (Kahn et al., 2014).

Once this qualitative argument is formulated mathematically, it can be generalized to any number of oscillators and equals the problem of evenly spacing interdependent onsets over time. Antisynchrony is its natural solution for two signallers. Among all possible phase relationships between oscillators, antisynchronous movements are *minimally* cluttered by others and occupy the sweet spot in time where no other animal has signaled, or will signal, for a whole *half period* (i.e., their onsets are evenly distributed and spaced in time, Figure 1H).

## Neural mechanisms underlying signal production, perception, and biases need not coincide

The neural mechanisms for *performing* and *perceiving* coordinated movements in humans and crabs are likely to differ (Hulse et al., 1984; Hulse and Kline, 1993; Harley et al., 2002; Hagmann and Cook, 2010; Hasegawa et al., 2011; Sztarker and Tomsic, 2011). Perception and production of rhythmic patterns seem to correlate with vocal learning across species (Patel, 2006, 2008; Patel et al., 2009; Schachner, 2010). Auditory and motor planning regions of the human cortex are linked more strongly than in many other species via dorsal auditory pathway connections (Patel and Iversen, 2014). This would explain the extreme flexibility some vocal learning mammals have in imitating new sounds by readily mapping perceived vocalizations into orofacial movements.

The neural bases of processing rhythmic information in crabs should be close to other arthropods. Common ancestry would suggest that crabs, like crickets or fireflies, use an ‘inflexible’ *phase-resetting* mechanism to time their movements (like turning a metronome off and on again). However, crabs appear more flexible than their insect relatives, decreasing the wave duration and between-wave pause the closer a female crab approaches (How et al., 2008). This offers initial support for the hypothesis that crabs might have a human-like *frequency modulation* mechanism (speeding up or slowing down, like a DJ mixing songs with different tempos). This hypothesis can be tested in fiddler crabs by varying the stimulus rate and adapting a suite of well-developed experimental paradigms (Repp, 2005; Repp and Su, 2013).

Several animals show antiphonal interactions (Ravignani et al., 2014b), which at least in a frog species (*Hyla japonica*) seem to reach the perfect alternation of antisynchronous calling (Aihara et al., 2014). However, group production of antisynchronous signals does not imply its perception. In turn, perceptual biases for a coordination pattern can only, although need not, emerge if a particular species already perceives that pattern.

## Future experiments across species: dynamical systems as roadmap to test the neuropsychology and genetics of perceived coordination

Perceptual antisynchrony is the first step to uncover the perception of coordination patterns across species. While systematic classification of interdependent temporal signaling in the animal kingdom is ongoing (Ravignani et al., 2014b), no common measure of (perceived) coordination complexity exists yet. Such measure would allow ranking different coordination patterns (synchrony, randomness, non-synchronous interdependence, etc.) along a neurobiological, perceptual dimension. The species tested until now seem to prefer synchrony, antisynchrony or both. Similarly, oscillators in synchrony and antisynchrony, although corresponding to the seemingly opposite phenomena of unison and alternation, are extremely close to each other in physical terms (Figures 1C,G). *Physical* measures of coordination complexity, as in dynamical systems (Winfree, 1986; Strogatz and Stewart, 1993; Strogatz, 2000; Large, 2008), might provide a valuable first approximation to *perceived* coordination.

Future behavioral research should test perception of different coordination patterns across species. Building on behavioral results, the long term goal will be to uncover the neuro-(epi)genetics (Lachmann and Jablonka, 1996; Petkov and Jarvis, 2012; Bronfman et al., 2014; Wilkins et al., 2014; Jablonka and Lamb, 2015) of temporal coordination. Recent evidence from musicians provides a first molecular and genetic link between joint coordinated actions and its perception, possibly transcending individual species. Researchers studied the genes transcribed after music performance (Kanduri et al., 2015a) and listening (Kanduri et al., 2015b), finding striking similarities with genes involved in song perception and production in songbirds. This suggests that some ancestral biological processes related to auditory-motor behavior, now crucial for song and speech, were preserved during 300 million years of independent evolutionary history (Kanduri et al., 2015a).^2^ Comparative research will enable mapping phylogenetic relations between species to the physical space of coordination patterns they perceive, hence unraveling the evolutionary history of those traits by homology or analogy (Tinbergen, 1963; Calvin, 1983; Ravignani et al., 2014a,b; Faunes et al., 2015).

## Funding

The author was supported by FWO grant V439315N (to Andrea Ravignani), and European Research Council grants 283435 ABACUS (to Bart de Boer) and 230604 SOMACCA (to W. Tecumseh Fitch).

### Conflict of interest statement

The author declares that the research was conducted in the absence of any commercial or financial relationships that could be construed as a potential conflict of interest.

## Footnotes

^1^This hypothesis of a bias toward the outcome of a group behavior differs from a simple precedence-effect bias toward one individual suggested elsewhere (cf. Reaney et al., 2008; Kahn et al., 2014).

^2^Perhaps few genetic commonalities underlie biases toward (anti)synchrony across species. Pending evidence from additional species, an antisynchrony bias in both humans and crabs is most parsimoniously (minimum number of gains and losses in a phylogenetic tree, cf. Petkov and Jarvis, 2012) explained by analogy.

## References

[B1] Aihara I. Mizumoto T. Otsuka T. Awano H. Nagira K. Okuno H. G.. (2014). Spatio-temporal dynamics in collective frog choruses examined by mathematical modeling and field observations. Sci. Rep. 4:3891. 10.1038/srep03891 24463569 PMC5384602

[B2] Backwell P. Jennions M. Passmore N. Christy J. (1998). Synchronized courtship in fiddler crabs. Nature 391, 31–32. 10.1038/34076

[B3] Bermejo M. Omedes A. (2000). Preliminary vocal repertoire and vocal communication of wild bonobos (*Pan paniscus*) at Lilungu (Democratic Republic of Congo). Folia Primatologica 70, 328–357. 10.1159/000021717 10640882

[B4] Bronfman Z. Z. Ginsburg S. Jablonka E. (2014). Shaping the learning curve: epigenetic dynamics in neural plasticity. Front. Integr. Neurosci. 8:55. 10.3389/fnint.2014.00055 25071483 PMC4083220

[B5] Brumm H. Slater P. (2007). Animal communication: timing counts. Curr. Biol. 17, R521–R523. 10.1016/j.cub.2007.04.053 17610836

[B6] Bryant G. A. (2014). The evolution of coordinated vocalizations before language. Behav. Brain Sci. 37, 549–550. 10.1017/S0140525X1300397X 25514939

[B7] Buck J. Buck E. (1968). Mechanism of Rhythmic Synchronous Flashing of Fireflies Fireflies of Southeast Asia may use anticipatory time-measuring in synchronizing their flashing. Science 159, 1319–1327. 10.1126/science.159.3821.1319 5644256

[B8] Buck J. (1988). Synchronous rhythmic flashing in fireflies. II. Q. Rev. Biol. 63, 265–287. 10.1086/415929 3059390

[B9] Calvin W. H. (1983). A stone's throw and its launch window: timing precision and its implications for language and hominid brains. J. Theor. Biol. 104, 121–135. 10.1016/0022-5193(83)90405-8 6632930

[B10] Cirelli L. K. Einarson K. M. Trainor L. J. (2014). Interpersonal synchrony increases prosocial behavior in infants. Dev. Sci. 17, 1003–1011. 10.1111/desc.12193 25513669

[B11] Dufour V. Poulin N. Curé C. Sterck E. H. (2015). Chimpanzee drumming: a spontaneous performance with characteristics of human musical drumming. Sci. Rep. 5:11320. 10.1038/srep11320 26080900 PMC4469965

[B12] Dunbar R. (2012). On the evolutionary function of song and dance, in Music Language, and Human Evolution, ed Bannan N. (Oxford, UK: Oxford University press), 201–214.

[B13] Ermentrout B. (1991). An adaptive model for synchrony in the firefly *Pteroptyx malaccae*. J. Math. Biol. 29, 571–585. 10.1007/BF00164052

[B14] Faunes M. Francisco Botelho J. Ahumada Galleguillos P. Mpodozis J. (2015). On the hodological criterion for homology. Front. Neurosci. 9:223. 10.3389/fnins.2015.00223 26157357 PMC4477164

[B15] Fitch W. T. Rosenfeld A. J. (2007). Perception and production of syncopated rhythms. Music Percept. 25, 43–58. 10.1525/mp.2007.25.1.43

[B16] Fitch W. T. (2009). The biology and evolution of rhythm: unraveling a paradox, in Language and Music as Cognitive Systems, eds Rebuschat P. Rohrmeier M. Hawkins J. A. Cross I. (Oxford, UK: Oxford University Press), 73–95.

[B17] Fitch W. T. (2015). Four principles of bio-musicology. Philos. Trans. R. Soc. Lond. B Biol. Sci. 370:20140091. 10.1098/rstb.2014.0091 25646514 PMC4321132

[B18] Fuhrmann D. Ravignani A. Marshall-Pescini S. Whiten A. (2014). Synchrony and motor mimicking in chimpanzee observational learning. Sci. Rep. 4:5283. 10.1038/srep05283 24923651 PMC5381545

[B19] Gamba M. Torti V. Bonadonna G. Guzzo G. Giacoma C. (2014). Overlapping and synchronization in the song of the Indris (Indri indri), in The Evolution of Language: Proceedings of the 10th international conference, eds Ea Cartmill S. R. Lyn H. Cornish H. (Singapore: World Scientific Press), 90–97.

[B20] Giacoma C. Sorrentino V. Rabarivola C. Gamba M. (2010). Sex differences in the song of Indri indri. Int. J. Primatol. 31, 539–551. 10.1007/s10764-010-9412-8

[B21] Grafe T. U. (1999). A function of synchronous chorusing and a novel female preference shift in an anuran. Proc. R. Soc. Lond. B Biol. Sci. 266, 2331–2336. 10.1098/rspb.1999.0927

[B22] Grahn J. A. Brett M. (2007). Rhythm and beat perception in motor areas of the brain. J. Cogn. Neurosci. 19, 893–906. 10.1162/jocn.2007.19.5.893 17488212

[B23] Greenfield M. D. Roizen I. (1993). Katydid synchronous chorusing is an evolutionarily stable outcome of female choice. Nature 364, 618–620. 10.1038/364618a0

[B24] Greenfield M. D. Tourtellot M. K. Snedden W. A. (1997). Precedence effects and the evolution of chorusing. Proc. R. Soc. Lond. B Biol. Sci. 264, 1355–1361. 10.1098/rspb.1997.0188

[B25] Hagen E. H. Bryant G. A. (2003). Music and dance as a coalition signaling system. Hum. Nat. 14, 21–51. 10.1007/s12110-003-1015-z 26189987

[B26] Hagen E. H. Hammerstein P. (2009). Did Neanderthals and other early humans sing? Seeking the biological roots of music in the territorial advertisements of primates, lions, hyenas, and wolves. Musicae Sci. 13, 291–320. 10.1177/1029864909013002131

[B27] Hagmann C. E. Cook R. G. (2010). Testing meter, rhythm, and tempo discriminations in pigeons. Behav. Process. 85, 99–110. 10.1016/j.beproc.2010.06.015 20600695

[B28] Haimoff E. H. (1986). Convergence in the duetting of monogamous Old World primates. J. Hum. Evol. 15, 51–59. 10.1016/S0047-2484(86)80065-3

[B29] Hall M. L. (2009). A review of vocal duetting in birds. Adv. Study Behav. 40, 67–121. 10.1016/S0065-3454(09)40003-2

[B30] Harley H. Odell K. Outnam E. Goonen C. Delong C. M. (2002). Dolphins perceive rhythm: a belated ode to Stewart Hulse, in 9th International Conference on Comparative Cognition (Melbourne Beach, FL).

[B31] Hartbauer M. Kratzer S. Steiner K. Römer H. (2005). Mechanisms for synchrony and alternation in song interactions of the bushcricket Mecopoda elongata (Tettigoniidae: Orthoptera). J. Comp. Physiol. A 191, 175–188. 10.1007/s00359-004-0586-4 15614532 PMC3971375

[B32] Hartbauer M. Römer H. (2014). From microseconds to seconds and minutes-time computation in insect hearing. Front. Physiol. 5:138. 10.3389/fphys.2014.00138 24782783 PMC3990047

[B33] Hasegawa A. Okanoya K. Hasegawa T. Seki Y. (2011). Rhythmic synchronization tapping to an audio-visual metronome in budgerigars. Sci. Rep. 1:120. 10.1038/srep00120 22355637 PMC3216601

[B34] Hattori Y. Tomonaga M. Matsuzawa T. (2013). Spontaneous synchronized tapping to an auditory rhythm in a chimpanzee. Sci. Rep. 3:1566. 10.1038/srep01566 23535698 PMC3610097

[B35] Hattori Y. Tomonaga M. Matsuzawa T. (2015). Distractor effect of auditory rhythms on self-paced tapping in Chimpanzees and humans. PLoS ONE 10:e0130682. 10.1371/journal.pone.0130682 26132703 PMC4488575

[B36] Hove M. J. Risen J. L. (2009). It's all in the timing: interpersonal synchrony increases affiliation. Soc. Cogn. 27, 949–960. 10.1521/soco.2009.27.6.949

[B37] How M. J. Hemmi J. M. Zeil J. Peters R. (2008). Claw waving display changes with receiver distance in fiddler crabs, Uca perplexa. Anim. Behav. 75, 1015–1022. 10.1016/j.anbehav.2007.09.004

[B38] Hulse S. H. Humpal J. Cynx J. (1984). Processing of rhythmic sound structures by Birdsa. Ann. N. Y. Acad. Sci. 423, 407–419. 10.1111/j.1749-6632.1984.tb23449.x 6588804

[B39] Hulse S. H. Kline C. L. (1993). The perception of time relations in auditory tempo discrimination. Anim. Learn. Behav. 21, 281–288. 10.3758/BF03197992

[B40] Inoue Y. Sinun W. Yosida S. Okanoya K. (2013). Intergroup and intragroup antiphonal songs in wild male MuellerÄôs gibbons (Hylobates muelleri). Interact. Stud. 14, 24–43. 10.1075/is.14.1.03ino

[B41] Iversen J. R. Patel A. D. Nicodemus B. Emmorey K. (2015). Synchronization to auditory and visual rhythms in hearing and deaf individuals. Cognition 134, 232–244. 10.1016/j.cognition.2014.10.018 25460395 PMC4255154

[B42] Jablonka E. Lamb M. J. (2015). The inheritance of acquired epigenetic variations. Int. J. Epidemiol. dyv020. [reprinted with permission from Jablonka, E., and Lamb, M. J. (1989). The Inheritance of acquired epigenetic variations. J. theor. Bioi. 139:69–83]. 10.1093/ije/dyv020 25855717

[B43] Janata P. Tomic S. T. Haberman J. M. (2012). Sensorimotor coupling in music and the psychology of the groove. J. Exp. Psychol. Gen. 141, 54. 10.1037/a0024208 21767048

[B44] Kahn A. T. Holman L. Backwell P. R. (2014). Female preferences for timing in a fiddler crab with synchronous courtship waving displays. Anim. Behav. 98, 35–39. 10.1016/j.anbehav.2014.09.028

[B45] Kanduri C. Kuusi T. Ahvenainen M. Philips A. K. Lähdesmäki H. Järvelä I. (2015a). The effect of music performance on the transcriptome of professional musicians. Sci. Rep. 5:9506. 10.1038/srep09506 25806429 PMC5380155

[B46] Kanduri C. Raijas P. Ahvenainen M. Philips A. K. Ukkola-Vuoti L. Lähdesmäki H.. (2015b). The effect of listening to music on human transcriptome. Peer J. 3:e830. 10.7717/peerj.830 25789207 PMC4362302

[B47] Keller P. E. Schubert E. (2011). Cognitive and affective judgements of syncopated musical themes. Adv. Cogn. Psychol. 7, 142–156. 10.2478/v10053-008-0094-0 22253676 PMC3259101

[B48] Kirschner S. Tomasello M. (2010). Joint music making promotes prosocial behavior in 4-year-old children. Evol. Hum. Behav. 31, 354–364. 10.1016/j.evolhumbehav.2010.04.004

[B49] Knoblich G. Butterfill S. Sebanz N. (2011). 3 Psychological research on joint action: theory and data. Psychol. Learn. Motiv. Adv. Res. Theory 54, 59 10.1016/B978-0-12-385527-5.00003-6

[B50] Kotz S. A. Schmidt-Kassow M. (2015). Basal ganglia contribution to rule expectancy and temporal predictability in speech. Cortex 68, 48–60. 10.1016/j.cortex.2015.02.021 25863903

[B51] Lachmann M. Jablonka E. (1996). The inheritance of phenotypes: an adaptation to fluctuating environments. J. Theor. Biol. 181, 1–9. 10.1006/jtbi.1996.0109 8796186

[B52] Large E. W. Gray P. M. (2015). Spontaneous tempo and rhythmic entrainment in a Bonobo (*Pan Paniscus*). J. Comp. Psychol.. [Epub ahead of print]. 10.1037/com0000011 26147705

[B53] Large E. W. (2008). Resonating to musical rhythm: theory and experiment. Psychol. Time 189–232. 10.1016/B978-0-08046-977-5.00006-5

[B54] Mann N. I. Dingess K. A. Slater P. (2006). Antiphonal four-part synchronized chorusing in a Neotropical wren. Biol. Lett. 2, 1–4. 10.1098/rsbl.2005.0373 17148310 PMC1617190

[B55] Manson J. H. Bryant G. A. Gervais M. M. Kline M. A. (2013). Convergence of speech rate in conversation predicts cooperation. Evol. Hum. Behav. 34, 419–426. 10.1016/j.evolhumbehav.2013.08.001

[B56] McNeill W. H. (1997). Keeping Together in Time. Cambridge, MA: Harvard University Press.

[B57] Merchant H. Zarco W. Pérez O. Prado L. Bartolo R. N. (2011). Measuring time with different neural chronometers during a synchronization-continuation task. Proc. Natl. Acad. Sci. U.S.A. 108, 19784–19789. 10.1073/pnas.1112933108 22106292 PMC3241773

[B58] Merker B. Madison G. S. Eckerdal P. (2009). On the role and origin of isochrony in human rhythmic entrainment. Cortex 45, 4–17. 10.1016/j.cortex.2008.06.011 19046745

[B59] Merker B. (2000). Synchronous chorusing and the origins of music. Musicae Sci. 3, 59–73. 10.1177/10298649000030S105

[B60] Miles L. K. Nind L. K. Macrae C. N. (2009). The rhythm of rapport: Interpersonal synchrony and social perception. J. Exp. Soc. Psychol. 45, 585–589. 10.1016/j.jesp.2009.02.002

[B61] Morris G. K. Kerr G. E. Fullard J. H. (1978). Phonotactic preferences of female meadow katydids (Orthoptera: Tettigoniidae: *Conocephalus nigropleurum*). Can. J. Zool. 56, 1479–1487. 10.1139/z78-205

[B62] Patel A. D. Iversen J. R. (2014). The evolutionary neuroscience of musical beat perception: the Action Simulation for Auditory Prediction (ASAP) hypothesis. Front. Syst. Neurosci. 8:57. 10.3389/fnsys.2014.00057 24860439 PMC4026735

[B63] Patel A. D. Iversen J. R. Bregman M. R. Schulz I. (2009). Experimental evidence for synchronization to a musical beat in a nonhuman animal. Curr. Biol. 19, 827–830. 10.1016/j.cub.2009.03.038 19409790

[B64] Patel A. D. (2006). Musical rhythm, linguistic rhythm, and human evolution. Music Percept. 24, 99–104. 10.1525/mp.2006.24.1.99

[B65] Patel A. D. (2008). Music, Language, and the Brain. New York, NY: Oxford University Press.

[B66] Petkov C. I. Jarvis E. D. (2012). Birds, primates, and spoken language origins: behavioral phenotypes and neurobiological substrates. Front. Evol. Neurosci. 4:12. 10.3389/fnevo.2012.00012 22912615 PMC3419981

[B67] Ravignani A. Bowling D. Kirby S. (2014a). The psychology of biological clocks: a new framework for the evolution of rhythm, in The Evolution of Language: Proceedings of the 10th International Conference, eds Ea Cartmill S. R. Lyn H. Cornish H. (Singapore: World Scientific Press), 262–269.

[B68] Ravignani A. Bowling D. L. Fitch W. T. (2014b). Chorusing, synchrony and the evolutionary functions of rhythm. Front. Psychol. 5:1118. 10.3389/fpsyg.2014.01118 25346705 PMC4193405

[B69] Ravignani A. Martins M. Fitch W. (2014c). Vocal learning, prosody, and basal ganglia: don't underestimate their complexity. Behav. Brain Sci. 37, 570–571. 10.1017/S0140525X13004184 25514960

[B70] Ravignani A. Olivera V. M. Gingras B. Hofer R. Hernández C. R. Sonnweber R.-S.. (2013). Primate Drum Kit: a system for studying acoustic pattern production by non-human primates using acceleration and strain sensors. Sensors 13, 9790–9820. 10.3390/s130809790 23912427 PMC3812580

[B71] Ravignani A. (2014). Chronometry for the chorusing herd: Hamilton's legacy on context-dependent acoustic signalling-a comment on Herbers (2013). Biol. Lett. 10:20131018. 10.1098/rsbl.2013.1018 24429686 PMC3917344

[B72] Reaney L. T. Sims R. A. Sims S. W. Jennions M. D. Backwell P. R. (2008). Experiments with robots explain synchronized courtship in fiddler crabs. Curr. Biol. 18, R62–R63. 10.1016/j.cub.2007.11.047 18211839

[B73] Repp B. H. Su Y.-H. (2013). Sensorimotor synchronization: A review of recent research (2006-2012). Psychon. Bull. Rev. 20, 403–452. 10.3758/s13423-012-0371-2 23397235

[B74] Repp B. H. (2005). Sensorimotor synchronization: a review of the tapping literature. Psychon. Bull. Rev. 12, 969–992. 10.3758/BF03206433 16615317

[B75] Ryan M. J. (1998). Sexual selection, receiver biases, and the evolution of sex differences. Science 281, 1999–2003. 10.1126/science.281.5385.1999 9748154

[B76] Schachner A. (2010). Auditory-motor entrainment in vocal mimicking species: Additional ontogenetic and phylogenetic factors. Commun. Integr. Biol. 3, 290–293. 10.4161/cib.3.3.11708 20714417 PMC2918780

[B77] Sismondo E. (1990). Synchronous, alternating, and phase-locked stridulation by a tropical katydid. Science 249, 55–58. 10.1126/science.249.4964.55 17787627

[B78] Strogatz S. H. Stewart I. (1993). Coupled oscillators and biological synchronization. Sci. Am. 269, 102–109. 10.1038/scientificamerican1293-102 8266056

[B79] Strogatz S. H. (2000). From Kuramoto to Crawford: exploring the onset of synchronization in populations of coupled oscillators. Physica D. 143, 1–20. 10.1016/S0167-2789(00)00094-4

[B80] Sztarker J. Tomsic D. (2011). Brain modularity in arthropods: individual neurons that support “what” but not “where” memories. J. Neurosci. 31, 8175–8180. 10.1523/JNEUROSCI.6029-10.2011 21632939 PMC6622864

[B81] ten Cate C. (2014). On the phonetic and syntactic processing abilities of birds: from songs to speech and artificial grammars. Curr. Opin. Neurobiol. 28, 157–164. 10.1016/j.conb.2014.07.019 25078891

[B82] Tinbergen N. (1963). On aims and methods of ethology. Zeitschrift für Tierpsychologie 20, 410–433. 10.1111/j.1439-0310.1963.tb01161.x

[B83] Trainor L. J. (2015). The origins of music in auditory scene analysis and the roles of evolution and culture in musical creation. Philos. Trans. R. Soc. Lond. B Biol. Sci. 370:20140089. 10.1098/rstb.2014.0089 25646512 PMC4321130

[B84] Tuttle M. D. Ryan M. J. (1982). The role of synchronized calling, ambient light, and ambient noise, in anti-bat-predator behavior of a treefrog. Behav. Ecol. Sociobiol. 11, 125–131. 10.1007/BF00300101

[B85] Wilkins A. S. Wrangham R. W. Fitch W. T. (2014). The “domestication syndrome” in mammals: a unified explanation based on neural crest cell behavior and genetics. Genetics 197, 795–808. 10.1534/genetics.114.165423 25024034 PMC4096361

[B86] Wiltermuth S. S. Heath C. (2009). Synchrony and cooperation. Psychol. Sci. 20, 1–5. 10.1111/j.1467-9280.2008.02253.x 19152536

[B87] Winfree A. T. (1986). Timing of Biological Clocks. Oxford, UK: Scientific American Library.

[B88] Yosida S. Kobayasi K. I. Ikebuchi M. Ozaki R. Okanoya K. (2007). Antiphonal Vocalization of a Subterranean Rodent, the Naked Mole-Rat (Heterocephalus glaber). Ethology 113, 703–710. 10.1111/j.1439-0310.2007.01371.x

[B89] Yosida S. Okanoya K. (2005). Animal cognition evolution of turn-taking: a bio-cognitive perspective. Cogn. Stud. 12, 153–165. 10.11225/jcss.12.153

[B90] Yu L. Tomonaga M. (2015). Interactional synchrony in chimpanzees: examination through a finger-tapping experiment. Sci. Rep. 5:10218. 10.1038/srep10218 25959242 PMC4426673

